# The Protest against Unemployment Insurance

**Published:** 1921-01-15

**Authors:** 


### THE PROTEST AGAINST UNEMPLOYMENT INSURANCE.

There is now no doubt at all that the general trend of nursing opinion runs strongly against unemployment insurance. The inquiry held by the Ministry of Labour on January 5, with Mr. B. O. Bircham in the Chair, was mainly limited to the question of whether sisters, nurses, and probationers employed in voluntary hospitals came within the scope of the Unemployment Insurance Act, but a certain latitude was observed in the discussion, and thus the general repugnance of all classes of nurses to the provisions made for their benefit in this Act became evident. The meeting was fairly representative. The opinion of some hospitals was voiced by their secretaries, and of nurses in hospital by Miss Cox Davies and a working sister. The College of Nursing, the Royal British Nurses' Association, Queen Victoria's Jubilee Institute, and the National Union of Trained Nurses were all represented, and the Chairman had therefore an opportunity of gauging the extent to which nursing interests opposed the measure. Miss Pearse, of the National Union of Trained Nurses, it is true, thought it possible that nurses might be profitably included under a special scheme which Section 18 gives power to the Minister to approve. On the other hand, 80 per cent. of the 3,000 nurses who gave in their opinion in reply to the College circular are unconditionally opposed to unemployment insurance for nurses, and this 80 per cent. does not include private nurses.

There are not wanting arguments which might appear to justify the Ministry of Labour in imposing such a measure upon persons reluctant to accept it. The Unemployment Insurance Act may claim to be a measure for the future rather than for the present, and the competence of nurses or their employers to prejudice the presumed interests of their successors in the profession may hence be called in question. It may be argued that the Ministry has better means of forecasting the result of unemployment insurance on any particular class of workers than these workers can themselves have access to. It may be argued that those who resist the benefits which it is proposed to confer by means of unemployment insurance are imitating the conduct of those boroughs which refused in the past century to allow railways to come within two miles of their towns, and that by such refusal they may hamper a generation yet unborn. We desire to give full weight to such a line of argument. But the onus of establishing what benefits will be conferred by a measure must ultimately rest on those who bring it in, and so far we have not seen any convincing argument in favour of extending this form of insurance to nurses. It is not a very plausible argument that a garment which at the present time is conspicuously ill-fitting will do well enough when the profession has, so to speak, grown to it. Unless here and now unemployment insurance is going to be of use to the nursing profession it is idle to contend that it will by and by.

The nurse's employment problems are of a very delicate nature. They depend partly on her physical fitness, but a great deal more on her moral and individual qualities. Obstinate cases of unemployment tend to reveal themselves as unemployableness. What are the administrators of the Unemployment Act going to do about such cases? There are unemployable nurses in every city. The only thing to do with them is to persuade them to embrace another calling. It can, however, be perceived that, under the insurance scheme, the Labour Bureaux would be mainly resorted to by women of an unsatisfactory type, whom they would continually be faced with the difficulty of placing out, not entirely to their own credit. There are plenty of nurses who are undesirable inmates of any private family or institution, though far from having actually lost their characters. These are the people who form the bulk of the unemployed nurse class.

Again, unemployed nurses, even when considerably pinched, are as a rule very far from desiring to accept the first post which offers, and it is quite possible that their chances of securing really suitable work might be prejudiced did they do so. If they are to be forcibly introduced to a system which will not work when they are in need of employment, they will be injured rather than benefited. The ordinary private nurse expects and intends to be unemployed for a certain time every year as a set-off to the hard times she goes through on duty.

It is perfectly clear that neither institution nor district nurses need ever be out of employment except when they are waiting for some special post, as regards locality or class of work. Whether this is a state of things which will always last none can say, but Miss Peterkin is convinced there need be no anxiety about unemployment for district nurses, at any rate for many years to come. In such circumstances the introduction of a complicated system which will cost one way and another a great deal of money can benefit at the best but a scanty minority, and at the worst could only inconvenience the majority. The case for further consideration is overwhelming. The absence of arguments in favour of the inclusion of institution nurses is actually startling.

